# Quackery

**Published:** 1845-07

**Authors:** 


### THE WESTERN JOURNAL.

New Series.—Vol. IV.—No. I.

LOUISVILLE, JULY 1, 1845.

### QUACKERY.

"Witches and impostors," says Bacon, "have always held a competition with physicians." No profession, trade, or calling, is so infested and overrun by quacks and pretenders as that of medicine. In every conceivable form, under every specious name, in every civilized country, quackery prevails; the people are willing to be deceived, and let them be deceived, is the maxim of the impostor. At no period in our world's history was the spirit of empiricism rifer than in this enlightened day. It would seem that men can become intelligent on all subjects but medicine. Concerning everything else they appear capable of reasoning, and can understand that other departments of human knowledge are attaining to higher perfection They have become persuaded that the lawyer who has studied his profession is a safer counsellor for his reading and experience, and that the blacksmith will shoe a horse better for having learned his trade; but in medicine learning passes with the multitude for nothing. The medical man of the people, like the '*medicine man*' of the Indians, and like Shakspeare's scholar, Dogberry, has his learning from "nature." Study cannot improve him. He is a man of one idea, and one remedy; but that remedy is sovereign, infallible, a veritable panacea. A thousand such *panaceas* are flaunted daily in the newspapers, and the credulous public read and believe the false promises, deceived every day, but ready after every disappointment to try some ever newer humbug.

We have been led into this train of reflection by a couple of documents which have lately come to us from different quarters. One of these is a pamphlet styled "*A Bomb in the Camp of the Enemy; or an exposure of Quackery and patent medicines,*" by Dr. Epperson, of Tennessee. The other is the second part of the sixth volume of the Transactions of the New York State Medical Society. Both of these publications refer much to the evils under which our profession labors, and especially to the growing evils of quackery.

We have had occasion to notice Dr. Epperson before, as an enlightener of the public on the subject of Human Health. His theme on the present occasion is a kindred one, and he handles it with his accustomed spirit. The following is a specimen of the manner in which he takes off the nostrum-mongers.

"Although these 'wonderful cures' have loudly threatened to annihilate the whole tribe of human maladies wherever they pass, yet we *see* no diminution of disease in their course. Look, for example, at consumption, that impudent '*wind-mill,*' in conflict with which the Don Quixotes of modern quackery have broken many a lance. If one-hundredth part of the professions of patent balsams, and syrups, and lozenges, etc., were half true, this naughty disease would not dare to show its head, for the war of extermination waged against it. But what is the fact? It not only exists, but is increasing the number of its victims. Not only so, but it presumes to 'carry the war into Africa.' For in the cities of New York and Philadelphia, where these terrible consumption-slayers are *forged* and swallowed in immense numbers, the disease is perhaps *more* fatal than in any other place. Surely then, if these boasted engines of war cannot defend their own cities from the attacks of the enemy, they ought not to be sent abroad to do battle."

But after all, the melancholy question comes up, *cui bono?* What good will all this exposure of quacks and quack medicines do? Few will read you, and yet fewer understand. Your motives will be misconceived; you are writing against patent medicines, it will be said, because you wish to sell your own. Our belief is that physicians cannot render their profession much ser vice by such a war against quacks. That "ineffable contempt" of which the author of this spicy pamphlet speaks at its close, is about as effectual as any remedy that can be applied to them.

In New York one of the so-called barriers to quackery has been broken down, and the last volume of Transactions issued by the Medical Society of the State, abounds in reflections on the condition and prospects of the profession in that great commonwealth. The Legislature has repealed the law requiring certain qualifications in the candidate for medical practice, "and the learned and the ignorant, the physician and the empiric now enter the arena even-handed to compete for the public favor." Such is the statement of the case, and at first sight the condition of things would strike one as rather forbidding; but the medical men who have written concerning it do not regard it as inauspicious. The law, they contend, had outlived its usefulness, and become a dead letter; its repeal, therefore, is not a source of regret with the profession. But some of them feel aggrieved by the course of a certain small gentleman, of the name of Scott, who, "dressed in a little brief authority," has availed himself of the privileges of his place to rail against a profession, of which it is presumed he is as ignorant, as he is of the common courtesies of life. Dr. Cook takes him sharply to task for his indecent attack, and holds him up to the scorn of all enlightened men. Forgetting the high station which it was the misfortune of the State that he should occupy, he seems to have "supposed himself pettifogging the case of some Homœopath or Thompsonian, in which the magnitude of the fee depended upon the extent to which he outraged truth." In this manner Dr. C. speaks of this legislator while proceeding to reply to his slanders.

Our brethren of the Empire State are making a noble effort in behalf of the profession. Their activity and zeal, as manifested in this volume of their Transactions, are worthy of the cause in which they are enlisted. They have a correct conception of the mode of suppressing quackery, which is to improve medical education, and give increased certainty to medical practice. Y.

